# Depression and Its Help Seeking Behaviors: A Systematic Review and Meta-Analysis of Community Survey in Ethiopia

**DOI:** 10.1155/2018/1592596

**Published:** 2018-12-18

**Authors:** Berhanu Boru Bifftu, Wubet Worku Takele, Yonas Deressa Guracho, Fekadu Ambaw Yehualashet

**Affiliations:** ^1^University of Gondar College of Medicine and Health Science, School of Nursing, Gondar, Ethiopia; ^2^Bahir Dar University college of Medicine and Health Science College Department of Psychiatry, Ethiopia

## Abstract

**Background:**

Depression is one of the most common mental illnesses affecting around 322 million individual in the world. Although the prevalence of depression is high and its treatment is effective, little is known about its pooled prevalence and help seeking behaviors in the community settings of Ethiopia. Thus, this study aimed to determine the pooled prevalence of depression and its help seeking behaviors in Ethiopia.

**Methods:**

A systematic literature search in the databases of Pub-Med, Cochrane, and Google Scholar was performed. The quality of studies was assessed using the Newcastle-Ottawa quality assessment tool adapted for cross-sectional studies. Heterogeneity test and evidence of publication bias were assessed. Moreover, sensitivity test was also performed. Pooled prevalence of depression and its help seeking behavior were calculated using random effects model.

**Results:**

A total 13 studies for depression, 4 studies for help seeking intention, and 5 studies for help seeking behaviour were included in this review. The pooled prevalence of depression and help seeking intention and behaviour was found to be 20.5% (95% CI; 16.5% -24.4%), 42% (95% CI; 23%-60%), and 38% (95% CI; 23%-52%), respectively. There is no significant heterogeneity for depression (I^2^ = 0%, p =0.620), help seeking intention (I^2^ = 0%, p =0.996), and behaviour (I^2^ = 0%, p =0.896). There is no publication bias for depression egger's test (p =0.689).

**Conclusion:**

More than one in every five individuals were experiencing depression. Less than one-third of individuals with depression seek help from modern treatment. Authors suggest community based mental health screening and treatment.

## 1. Background

Mental illness is a growing concern of public health that affects more than 340 million people worldwide. Out of the top 10 leading causes of disability, five are mental illnesses [1–3]. Depression is one of the most common mental illnesses with an estimated prevalence of 4.4% globally [2]. The prevalence of depression is on rising by 18.4% from 2005 to 2015 [3]. Depression increased the mortality, morbidity, disability, family, and the country as whole [1, 2, 4, 5]. Depression also affects occupational and interpersonal functioning of the individual [6–8], particularly the cost associated with loss of working days [6]. Moreover, depression increases the risk of cardiac illness, diabetes, and hypertension [6, 9]. In Ethiopia, depression is the third leading cause of burden of diseases [7]. The magnitude and the adverse impact of depression are unbearable [10–12] with an estimated prevalence ranging from 2.4% [13] to 60% [10, 14–27]. It contributed as a third leading cause of burden of diseases [7]. It also contributes for the comorbid medical [28–34] and surgical illness and affect the treatment adherence [35–38]. Moreover, depression contributes for substance use [17, 39] and suicide [8, 40, 41]. Factors such as low socioeconomic status, residence, sex, substance use, previous history of mental illness, family history of mental illness, and chronic illness were associated with depression [15, 17, 24, 30, 31, 42].

Depression is a treatable mental illness [1, 2, 12, 43]. Despite its huge impact and availability of effective treatment [1, 43–47], vast majority of individuals who suffer from mental illness do not access treatment for their problems. In general, epidemiological evidence revealed the prevalence of professional help seeking behavior for mental illness is ranging from 35% to 50% in developed countries [43, 48] and from 10% to 15% in developing countries [12, 42, 44, 49–51]. In regard to depression, the professional help seeking behavior was ranging from 33% in Africa to 55.6% in Europe [52] with a regional variation [20, 53, 54]. Factors such as fear of stigma and embarrassment, lack of time, comorbid substance use, demographic and geographical variation [50], co-morbid chronic illness [49, 55], psychosocial factors [5, 43, 56–58], perceived cause, accessibility, and effectiveness of the treatment [59, 60] were associated with the treatment gap. In Ethiopia, the treatment gap for mental illness reached 90% [12, 27, 61–64], and specifically for depression, it ranged from 12% to 82% [18, 20, 27]. Communities' knowledge, attitude, substance use, socioeconomic and geographic factors [12, 65, 66], lack of resource, and the dominance of traditional healer were barrier for the treatment seeking behaviors [67, 68]. Untreated depression is a potential for symptoms of severe treatment resistant and increased burden and complexity of the disorders [7, 41, 69, 70]. Therefore, studying the help-seeking behavior enables us to understand and discover people's dynamics in their decisions of help seeking behavior from trained health care professional. Moreover, it gives vital information on communities' attitude and practice toward their preference treatment place for depression. This information helps the planning and provision of training to strengthen the referral mechanisms and health policy of the country to address the choice of the communities' treatment place. In Ethiopia, there is inconsistent epidemiological evidence about depression and its help seeking behavior [18, 20, 27], which need comprehensive evidence for decision making and, yet, there are no a single systematic review and meta-analysis using community survey. Thus, the purpose of this systematic review and meta-analysis was to assess the magnitude of depression and its help seeking behaviors in Ethiopia.

## 2. Methods

We conducted a systematic review and random effects meta-analysis to identify the magnitude of depression and its help seeking behaviors using the Preferred Reporting Items for Systematic Review and Meta-Analysis (PRISMA) [71].

### 2.1. Search Strategies

We searched databases: PubMed, Cochrane, and Google Scholar. PubMed electronic database was searched until September, 15, 2018, using the search term ((depression [MeSH Terms]) OR (depression) OR (depressive symptoms [MeSH Terms]) OR (depressive symptoms) OR (depressive disorders [MeSH Terms]) OR (depressive disorders) OR (dysthymia [MeSH Terms]) OR (dysthymia) OR (mood disorder [MeSH Terms]) OR (mood disorder)) AND ((disclosure [MeSH Terms]) OR (disclosure) OR (coping mechanism [MeSH Terms]) OR (coping mechanism) OR (defense mechanism [MeSH Terms]) OR (defense mechanism) OR (resilience [MeSH Terms]) OR (resilience) OR (health care utilization [MeSH Terms]) OR (health care utilization) OR (health service utilization [MeSH Terms]) OR (health service utilization) OR (help seeking behavior [MeSH Terms]) OR (help seeking behavior) OR (help seeking intention [MeSH Terms]) OR (help seeking intention OR (treatment seeking [MeSH Terms]) OR (treatment seeking) OR (health care seeking behavior [MeSH Terms]) OR (health care seeking behavior) OR (healer [MeSH Terms]) OR (healer) OR (pathway [MeSH Terms]) OR (pathways) OR (services contact [MeSH Terms]) OR (service contact) OR (first contact [MeSH Terms]) OR (first contact) OR (help seeking [MeSH Terms]) OR (help seeking)) AND Ethiopia. There was no restriction on language and year of publication. The reference lists of included studies were manually searched. Likewise, Cochrane review databases were searched using similar search terms tailored to it. Google Scholar was also searched for gray literature and published paper in unindexed journals. For the required information not clear/not avail, authors were contacted via email.

### 2.2. Selections of the Studies

**Figure 1:**
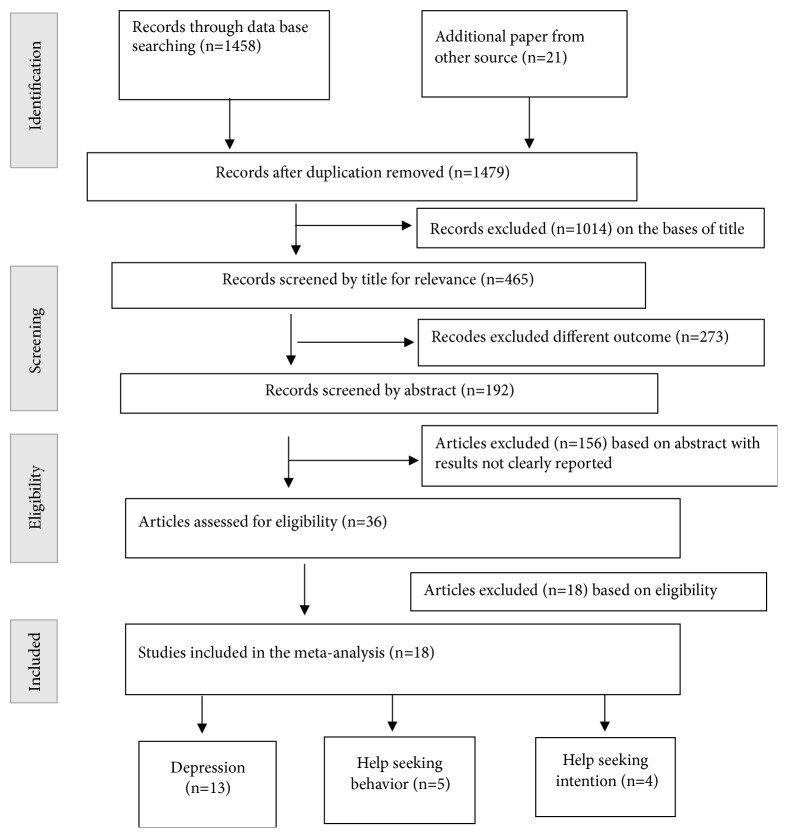
Flow diagram of the included studies.

Figure 1 showed the study selection process. All citations (*N *= 1458) identified through our search strategy were imported into EndNote version X7 reference management software and used automated “Find Duplicates” function to exclude any duplicates. The title and abstracts of the 1458 articles were assessed by two reviewers (BBB and YDG).

### 2.3. Definition of the Variables

In this systematic review and meta-analysis, depression refers to the occurrence of either depression (major or minor), depressive symptom, dysthymia, or their combinations. Help seeking behavior is an individual's willingness/intention to seek help or actual help seeking behavior from at least one of modern health facilities such as: hospital, health center, clinic, health post and/or from health care professional (psychiatrist, nurse, psychologist, sociologist and other) and coded as “Yes”/“No.”

### 2.4. Inclusion and Exclusion Criteria

#### 2.4.1. Participants

They include any population based study including those on behalf of another individual (e.g., family members).

They exclude homeless, livening abroad, specific group (e.g., women, people with disability), and refugee.

#### 2.4.2. Outcomes

Studies presenting quantitative data on (i) depression and (ii) help seeking behavior for depression or depressive symptom from modern treatment place (hospital, health center, clinic, and health post) and or health care professionals (psychiatrist, psychologist nurse, and others) and coded as “Yes”/“No” for analysis.

Depression with comorbid medical or surgical illness, substance use, and other psychiatric illnesses are excluded.

Help seeking for other mental illness (psychosis, suicide and substance use disorder, and others).

#### 2.4.3. Study Designs

Community based quantitative cross-sectional studies design is included.

Institution based qualitative study design that did not estimate the prevalence of depression and help seeking behavior is excluded. Moreover, studies that focus on case reports, paper, and conference abstracts that did not provide enough information were excluded.

*Data Extraction*. A standardized and prepiloted checklist was used to extract the required information. Data were extracted on study characteristics and outcomes by two independent reviewers (BBB and YDG) and stored in a Microsoft Excel Spread Sheet. The extracted data include details of author's name, year of publication, study area, sample size, assessment tool, prevalence of depression, and help seeking behaviors (intention and actual help seeking behavior).

### 2.5. Quality Assessment

Two review authors' independently assessed the quality of included studies using the Newcastle-Ottawa quality assessment tool adapted for cross-sectional studies. This tool had seven items in three domains (selection, comparability, and outcome). Individual paper was grade with score ranged from zero to nine. Quality of each paper was determined using the overall sum of each item score and defined as good for score ≥5 and fair for score ranged from 3 to 4 and poor for score <3. This quality appraisal score was assessed by two investigators (BBB and YDG) and disagreements were solved by discussion.

### 2.6. Data Synthesis and Statistical Analysis

The extracted data were entered into a Microsoft Excel Database and then imported into STATA 14 that we installed packages for Meta-analyses online. Meta-analyses were performed separately for each outcome: depression, help seeking behavior (help seeking intention and actual help seeking behavior). The estimated pooled prevalence and weighted mean differences of depression and help seeking behavior were calculated using random-effects model at 95% confidence interval [72]. Test for Heterogeneity between the studies was performed using Cochran's Q statistic and the* I*^2^ statistics [73]. I^2^ values greater than 50% were considered as indicative of substantial heterogeneity. Evidence of publication bias was assessed using visual inspection of the symmetry in funnel plot [74] and egger test [75]. Sensitivity analysis was also conducted to examine influential study [76].

## 3. Results

The literature search resulted in 1458 recorded papers. Of these records, 1014 were excluded just by reading their titles. Of the remaining 273 studies, 156 were excluded by reading their abstract and found to have a different outcome variable. Finally, 18 papers were excluded because of their different methods and prevalence/results were not clearly reported. Thus, the remained 18 studies were included in the systematic review and meta-analysis (Figure 1).

### 3.1. Study Characteristics

A total of 13 studies for depression, 4 studies for help seeking intention, and 5 studies for help seeking behaviour were included in this meta-analysis. All studies were community based and utilized cross-sectional study design. Of these included studies, two studies were from Amhara, three studies were from Oromia, two studies were from Southern Nation and Nationalities of People, and one study was national data. For the assessment of depression PHQ-9 (n=4), ICD-10 (n=2), DSM-IV (n=3), BDI-21 (n=1), HSCL-15 (n=1), and SCAN (n=1) were utilized. One study also reported use of the WHO STEPS instruments. For the assessment of help-seeking behavior case vignette (n=7) and GHSQ (n=2) were used (Table 1).

### 3.2. Quality of Included Studies

The overall quality score of included studies ranged from 3 to 8. Of these, 14 studies had good quality and the remaining 4 studies had poor quality (Table 2).

### 3.3. Depression

**Figure 2:**
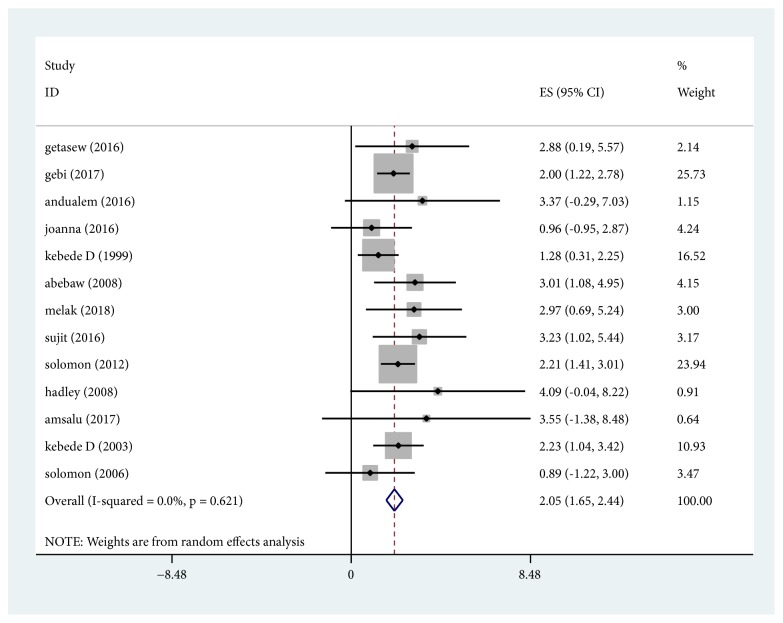
Forest plot presenting the pooled prevalence of depression using random effect models with 95% CI.

A total of 13 studies, including data from 20067 participants, were included in the study. The pooled prevalence of depression was found to be 20.5% (95% CI; 16.5% -24.4%) (Figure 2). There is no evidence of significant heterogeneity (I^2^ = 0%, p =0.620) and publication bias egger's test (p =0.637). The sensitivity analysis showed that none of the point estimates was outside of the overall 95% confidence interval confirming that there was no influential study. Thus, pooled estimates based on all the 13 studies could be important.

### 3.4. Help Seeking for Depression

**Figure 3:**
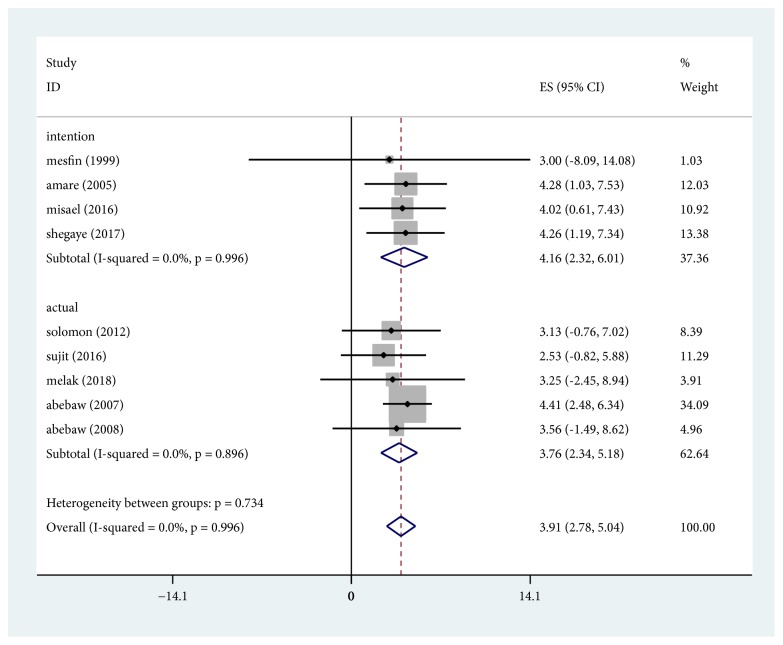
Forest plot presenting the help seeking intention and behaviour using random effect models with 95% CI.

In this systematic review and meta-analysis, the pooled prevalence of help seeking intention and behaviour was found to be 42% (95% CI; 23%-60%) and 38% (95% CI; 23%-52%), respectively (Figure 3). No evidence of significant heterogeneity help seeking intention (I^2^ = 0%, p =0.996) and behaviour (I^2^ = 0%, p =0.896). The overall sensitivity analysis showed that none of the point estimates was outside of the overall 95% confidence interval confirming that there was no influential study. Although evidence from visual inspection of the funnel plot and the Egger's test (P =0.025) showed publication bias, there is no change in the trim and fill analysis.

## 4. Discussion

The current review is the only community based epidemiological review of depression and its help seeking behaviors in Ethiopia. More than one in five of the community reside were found to have depression in Ethiopia [20.5% (95% CI; 16.5% -24.4%)]. This study confirmed the existence of high magnitude of depression in Ethiopia. Compared to other studies carried out by World Mental Health Survey Initiative at different time [(4.1/9.8% to 19.1% for the last 12 months) and (18.1% to 36.1% for the lifetime prevalence)] [77–79], we found higher prevalence of depression. This study also higher than a recently published community based samples of meta-analysis over one year (12.9%) [80]. This variation may be due to the difference in study period. This is supported by the results of subgroup analysis that showed the prevalence of depression in studies published from 2004 to 2014 was higher than those published from 1994 to 2003 [80]. The other possible explanation may be due to the regional variation [78, 79]. As supported by the WMHS epidemiological variation of depression across country that may attributed to the variation in the concept of the illness, socio-economic status and psychosocial norm.

Regarding the help seeking behaviors, the pooled prevalence of help seeking intention was found to be 48% (95% CI; 38%-57%). This may be an indication for mental health treatment gap in Ethiopia. Even though, similar studies are scarce for comparison this study, there are little supportive studies from developed and developing countries. This result is within the range of (49% to 84%) of other review on perceived need for treatment [81]. Other review of public study also found the treatment recommendation for depression was 49% (48%-50%) for medication and 76% (76%-77%) for psychotherapy [82]. The public's beliefs about the helpfulness of interventions actually influence their service use for mental health problems is important. On the other hand, even though 42% (95% CI; 23%-60%) of our study showed help seeking intention, the pooled actual help seeking behaviour was found to be low [38% (95% CI; 23%-52%)]. This suggests the needs of assessment that interfered with the actual use of the preferred treatment. This showed all individuals who willing may not perform the actual behaviours. This is supported by a study which showed that, out of 80% adults who agreed (slightly or strongly) that treatment can help people living with mental illness lead normal lives, 35% to 67% adults agreed that other people are caring and sympathetic to people living with mental illness. People's beliefs toward individual with mental illness usually affect interacting with, providing opportunities, and supporting a person with mental illness and frame whether they disclose the symptoms and seek care [83]. Compared to other systematically reviews, this result is consistent with the WHO African region study which showed 33% of treatment seeking behavior for depression [52]. Another systematic review also revealed a treatment-seeking behavior ranging from 17.0% and 77.8% of treatment [84]. A recently published WHO survey showed overall 29.0% of treatment [50]. This is also supported by a review from Africa using a pathways to care [50, 85] with an estimated pooled proportion ranging from 38% to 60.4% first treated by professionals [86]. Thus, the results of this finding implies though limited studies are avail: (i) the need of more study and (ii) high magnitude of depression among the Ethiopia community, (iii) with low help seeking behavior. This may suggest the needs of more information and evaluate the effectiveness of the current mental health care system on the screening of depression and its help seeking behavior from modern treatment place/professionals.

### 4.1. Strengths and Limitations of the Study

To our knowledge, this is the first systematic review and meta-analysis about the epidemiology of depression and its help seeking behavior in Ethiopia. Moreover, there are inclusions of all studies without the restrictions of year of publication and language. In addition to this, we conducted heterogeneity test and sensitivity analysis for possible sources of heterogeneity and identified influential paper across the studies. However, some limitations are considered during the interpretation of the results. First, although the focus of this study was on quantitative studies, the exclusion of qualitative studies and institution based studies minimize the number of included studies. Second, although we used reference lists and Google Scholar to include all the available studies, there may be possibility of having some overlooked articles. Finally, the use of case vignettes in the studies that may have different concept in the community recognitions of the disorder and help seeking behavior minimize the true figure. Despite these, this systematic review and meta-analysis revealed the recently available evidence that help to narrow the scant studies in the area, particularly in Ethiopia for the development of and/or strengthening the mental health care needs of the community.

### 4.2. Conclusion and Recommendations

More than one among five individuals were experiencing depression and less than one-third of individuals with depression seek help from modern treatment. Thus, authors suggest the need of community based mental health care for early identification and provisions of accessible, affordable, and cost effective treatment.

## Figures and Tables

**Table 1:** Characteristics of included studies.

Author Year	Setting	Design	Outcome	Sample size	Number of cases	Tool
Mulatu, 1999	Amhara	CS	Intention	50	10	Case vignette

Derbew, 2005	Oromia	CS	Intention	728	527	Case vignette

Benti, 2016	Oromia	CS	Intention	816	454	Case vignette

Hailemariam, 2012	NHS	CS	Depression	4925	449	ICD-10
			Actual	449	103	Case vignette

Rathod, 2016	SNNP	CS	Depression	1489	375	PHQ-9
			Actual	375	47	Case vignette

Menberu, 2018	Amhara	CS	Depression	1665	226	PHQ-9
			Actual	226	58	GHSQ

Sumet, 2017	Amhara	CS	Intention	832	592	GHSQ

Fekadu, 2007	SNNP		Actual	1511	1242	Case vignette

Fekadu, 2008	SNNP	CS	depression	1673	341	DSM-IV
			Actual	343	121	Case vignette

Molla, 2016	Amhara		Depression	779	139	PHQ-9

Hussien, 2017	Oromia		Depression	4371	323	SELF

Mossie, 2016	Oromia		Depression	590	171	BDI-21

Bartlett, 2016	Amhara		Depression	268	7	ICD-10

Kebede, 1999	AA		Depression	1420	51	HSCL-15

C. Handeley, 2008	SNNP		Depression	541	324	DSM-IV

Taye, 2017	Oromia		Depression	359	125	SCAN

Kebede, 2003	Oromia		Depression	2285	212	DSM-IV

Hailemariam, 2006	Oromia		Depression	205	5	ICD-10

***Note.***GHSQ**:** general help-seeking questionnaire, HSCL: Hopkins Symptoms Checklist, SNNP: Southern Nation and Nationalities of People, PHQ-9: Patient Health Questionnaire-9, CES-D: Center for Epidemiological Studies Depression Scale, BDI-21: Beck Depression Inventory, ICD-10: International Composite Diagnostic, SCAN: Schedule for Clinical Assessment in Neuropsychiatry, and DSM-IV: diagnostic and statistical manual of mental disorders.

**Table 2:** Quality of included studies in the analysis.

**Author, year**	**Quality domain**							**Overall score**
	**Selection**				**Comparability**	**Outcome**		
	**(Max score=5)**				**(Max=2)**	**(Max=3)**		

	(1) Representativeness of the sample: (a) Truly representative of the average in the target population*∗* (all subjects or random sampling) (b) Somewhat representative of the average in the target population*∗* (non-random sampling) (c) Selected group of users. (d) No description of the sampling strategy.	(2) Sample size: (a) Justified and satisfactory*∗* (b) Not justified.	(3) Non-respondents: (a) Comparability between respondents and non-respondents characteristics is established, and the response rate is satisfactory*∗* (b) The response rate is unsatisfactory, or the comparability between respondents and non-respondents is unsatisfactory. (c) No description of the response rate or the characteristics of the responders and the non-responders.	(4) Ascertainment of the exposure (risk factor): (a) Validated measurement tool. *∗∗* (b) Non-validated measurement tool, but the tool is available or described.*∗* (c) No description of the measurement tool.	(1) The subjects in different outcome groups are comparable, based on the study design or analysis. Confounding factors are controlled. (a) The study controls for the most important factor (select one)*∗* (b) The study control for any additional factor*∗* (c) no control	(1) Assessment of outcome (a) Independent blind assessment *∗∗* (b) Record linkage*∗∗* (c) Self report *∗* (d) No description.	(2) Statistical test: (a) is clearly described, appropriate, & measurement of association is presented, including confidence intervals & probability level (p value)*∗* (b) is not appropriate	

Mesfin, 1999	b (+1)	b(+0)	c (+0)	b (+1)	a(+1)	c (+1)	a(+1)	5

Derbew, 2005	a (+1)	a(+1)	a (+1)	b (+1)	a (+1)	c (+1)	a(+1)	7

Misael, 2016	b (+1)	a(+1)	a(+1)	b (+1)	a(+1)	c (+1)	a(+1)	5

Solomon, 2012	b (+1)	b(+0)	c(+0)	c (+0)	c (+0)	c (+1)	a(+1)	3

Abebaw, 2008	b (+1)	b(+0)	c(+0)	c (+0)	c (+0)	c (+1)	a(+1)	3

Abebaw, 2007	b (+1)	b(+0)	c(+0)	c (+0)	c (+0)	c (+1)	a(+1)	3

Suijit, 2016	b (+1)	b(+0)	a (+1)	b(+1)	a (+1)	c (+1)	a(+1)	6

Melak, 2018	b (+1)	a(+1)	a (+1)	a (+2)	a (+1)	c (+1)	a(+1)	8

Shegaye, 2017	b (+1)	b(+1)	b (+1)	a (+2)	a (+1)	c (+1)	a(+1)	8

Getasew, 2016	a (+1)	a(+1)	a (+1)	b (+1)	a (+1)	c (+1)	a(+1)	7

Gibi, 2017	a (+1)	a(+1)	a (+1)	c (+0)	a (+1)	c (+1)	a(+1)	6

Andualem, 2016	a (+1)	a(+1)	a (+1)	b (+1)	a (+1)	c (+1)	a(+1)	7

Joanna, 2016	a (+1)	a(+1)	a (+1)	b (+1)	a (+1)	c (+1)	a(+1)	7

Kebede, 1999	a (+1)	a(+1)	a (+1)	b (+1)	a (+1)	c (+1)	a(+1)	7

Handeley, 2008	a (+1)	a(+1)	a (+1)	b (+1)	a (+1)	c (+1)	a(+1)	7

Amsalu, 2017	a (+1)	a(+1)	a (+1)	b (+1)	a (+1)	c (+1)	a(+1)	7

Kebede, 2003	a (+1)	a(+1)	a (+1)	b (+1)	a (+1)	c (+1)	a(+1)	7

Solomon, 2006	a (+1)	a(+1)	c(+0)	b (+1)	a (+1)	c (+1)	a(+1)	6

## Data Availability

All data are included in this paper.

## Supplementary Materials

Supplementary Materials PRISM CHECK LIST for the systematic review and meta-analysis of CMDs among women with obstetric fistula.
